# Using event-related potentials to measure phrase boundary perception in English

**DOI:** 10.1186/s12868-014-0129-z

**Published:** 2014-11-26

**Authors:** Varghese Peter, Genevieve McArthur, Stephen Crain

**Affiliations:** ARC Centre of Excellence in Cognition and its Disorders, Department of Cognitive Science, Macquarie University, Sydney, Australia; MARCS Institute, University of Western Sydney, Locked Bag 1797, Penrith, NSW 2751 Australia

**Keywords:** Phrase boundary, Closure positive shift, Speech

## Abstract

**Background:**

The closure positive shift (CPS) event related potential (ERP) is commonly used as a neural measure of phrase boundary perception in speech. The present study investigated whether the CPS was elicited by acoustic cues at phrase boundaries in English. ERPs were recorded when participants listened passively to sentences with either early or late phrase boundaries.

**Results:**

The closure positive shift (CPS) ERP was elicited at both early and late phrase boundaries. However, the latency, amplitude, and scalp distribution of these passive CPS ERPs in English sentences differed to active CPS ERPs measured in non-English sentences in previous studies.

**Conclusions:**

These results show that acoustic cues at the phrase boundaries in English are sufficient to elicit the CPS, and suggest that different processes might be involved in the generation of the CPS in active and passive conditions.

## Background

Most adults understand the speech of other people with little apparent effort. This is an amazing feat given the complexity of the speech signal, which contains a continuous stream of words that are seldom separated by perceptible gaps. To make the meaning of a word-stream clearer to a listener, the speaker groups the words into separate meaningful phrases [1]. In English, the end of each phrase - the phrase boundary - is marked by acoustic cues, such as lengthening the final syllable [2], rise or fall in the fundamental frequency of the speech [3,4], and by inserting a pause after the final syllable [5].

Phrase boundaries aid speech comprehension in at least two ways. First, phrase boundaries promote semantic processing. The sentence “*Rob or Sandra and Mary will come”* has different meanings depending on whether a phrase boundary is placed after *“Rob”* or after *“Sandra”.* Second, phrase boundaries can define grammatical boundaries in speech. For example, in the sentence “*The little girl over there, is the one who likes snails*”, a careful speaker of the language will insert a phrase boundary between the words “*there*” and “*is*”. By dividing the sentence into two meaningful chunks - a noun phrase and a verb phrase – the speaker clarifies the syntax of the language [6-8].

Many behavioural studies have investigated the importance of phrase boundaries for the comprehension of language (for a detailed review, see [9]). Most of these studies have focused on how phrase boundaries resolve syntactic and semantic ambiguities, and hence have presented listeners with atypical sentences that contain violations in syntactic or semantic structure. Unfortunately, these studies cannot tell us how phrase boundaries are processed in typical sentences that do not have structural violations [10].

Fortunately, a handful of event-related potential (ERP) studies have investigated the processing of phrase boundaries in normal sentences. ERPs reflect the average pattern of electrical activity generated by large groups of brain cells in response to a particular sensory or cognitive event [11]. This electrical activity can be detected by small metal sensors which are placed on the scalp. Since ERPs are a direct measure of the electrical activity in the brain, they reflect processing in almost real time. Thus, ERPs can be used to measure the temporal processing of phrase boundaries in normal sentences.

Steinhauer, Alter and Friederici [12] were the first to report an ERP component that could be elicited by phrase boundaries in normal sentences. They presented listeners with German sentences with either one *(“Peter promises Anna to work # and to clean the office”*) or two *(“Peter promises # to support Anna # and to clean the office”*) phrase boundaries (# indicates phrase boundary). In 20% of trials, the listeners were asked a yes/no question at the end of the sentence. The “active” ERP (i.e., the ERP measured while listeners actively attended the stimuli) showed a positive shift in the electrical activity around 500 ms after the phrase boundary, which was largest at central and parietal scalp sites. Since the ERP component showed a positive shift in electrical activity at the closure of the phrase, the component was called closure positive shift (CPS).

Steinhauer and Friederici [13] investigated whether the CPS was related to acoustic or linguistic cues at phrase boundaries. They presented listeners with normal and delexicalised sentences (i.e., low-pass filtered sentences with no linguistic cues) that had one or two phrase boundaries. For normal sentences, listeners were asked a yes/no question about the meaning of the sentence. The delexicalised sentences were followed by visual presentation of a normal sentence, and listeners were instructed to recreate the melody of the delexicalised sentence while reading the normal sentence. A CPS was seen at phrase boundaries in the normal sentences, and at the first phrase boundary in the delexicalised sentences. From this, the authors concluded that the CPS is related to the acoustic cues that mark phrase boundaries, rather than linguistic cues. However, the figures in this study showed that the CPS in the delexicalised sentences was different in shape and amplitude to the CPS elicited by the normal sentences. Also, no CPS was seen at a second phrase boundary for delexicalised sentences. This suggests that the CPS is not the result of acoustic cues alone. Instead, both acoustic and linguistic cues elicit the CPS at phrase boundaries in normal sentences.

Pannekamp et al. [10] furthered this work by presenting adults with four types of sentences with either one or two phrase boundaries: normal sentences, jabberwocky sentences (i.e., content words were replaced by meaningless words that lacked semantic information), pseudo sentences (i.e., content words and function words were replaced by meaningless words that lacked syntactic and semantic information) and hummed sentences (i.e., the intonation contour of the sentences were hummed and hence had no syntactic, semantic or phonemic content). After each sentence, listeners were asked to decide whether a probe word was a part of the sentence. A CPS was present in all the conditions. However, the topography of the CPS (i.e., the pattern of electrical activity across the scalp) differed between sentences. For normal sentences, a CPS was seen at both central and lateral sites. As the linguistic cues were reduced (i.e., syntactic, semantic and phonemic information removed), the CPS was lateralised to anterior locations in the right hemisphere. This again suggests that both acoustic and linguistic cues elicit the CPS in normal sentences.

The degree to which acoustic and linguistic cues contribute to the CPS has yet to be established. To date, the majority of CPS studies support the idea that acoustic prosodic cues are primarily responsible for the generation of CPS (though cf. [14]), while linguistic cues modulate its amplitude and scalp topography [15]. However, most of these studies have been done in German [10,12,13,16-21], with a handful in Swedish [22], Korean [23], Dutch [24,25], Mandarin [26], and Japanese [27]. This is problematic for understanding the CPS in English because phrase boundarires are marked by different acoustic cues in different languages. For example, phrase boundaries are marked by changes in duration and intensity in German [28] but by changes in duration, pitch and pause in English [2,3,5]. This raises the question of whether acoustic cues for phrase boundaries elicit a CPS in normal English sentences.

The role of acoustic cues in the generation of CPS in English has been addressed in part by Itzhak et al. [29]. They investigated acoustic phrase boundaries and transitivity bias on ERPs in English speakers. Transitivity bias is the probability that a verb will be followed by an object (e.g. *“He lifted the bag”* versus *“She slept”*). They presented listeners with sentences that either had a high transitively biased verb or a low transitively biased verb. In half the sentences, the verb was followed an acoustic phrase boundary (e.g. *While Billy was playing # the game seemed simple*). Participants were asked to make an acceptability judgement after each sentence. The authors found a front-central CPS between 150–300 ms for the sentences with a phrase boundary, and a CPS-like positivity in sentences without a phrase boundary when the verb was highly transitively biased. They concluded that the high transitivity of the verb was sufficient to drive the brain systems to impose a boundary after the verb despite the absence of any acoustic phrase boundary.

The presence of a CPS in the absence of acoustic cues in Itzhak et al. [29] suggests that acoustic cues for phrase boundaries are not a major generator of the CPS. However, the strength of this suggestion is limited by the fact that this study did not measure the CPS to acoustic cues alone. To determine if acoustic cues elicit a CPS in English, we need to measure the CPS in normal sentences in a way that minimises the processing of linguistic cues (i.e., syntax and semantics). This might be done with passive ERPs, which are ERPs measured while a subject’s attention is diverted away from the stimulus of interest (e.g., to a computer game or movie). Thus, the aim of this study was to use passive ERPs to test, for the first time, if acoustic cues for phrase boundaries elicit a CPS in normal English sentences in adults.

## Method

### Ethics statement

The Ethics Committee for Human Research at Macquarie University approved the experimental methods used in this study (approval number: HE23NOV2007-D05579). Written informed consent was obtained from all the participants.

### Subjects

Twenty-four paid volunteers participated in the study (13 female; mean age: 22.38 years; *SD*: 2.42). All the subjects were native speakers of English, passed a hearing screening for both the ears (hearing thresholds within 20 dB HL for 500 Hz, 1 kHz, 2 kHz and 4 kHz in both ears), and were strongly right handed as measured by Edinburgh Handedness Inventory [30]. Data from one additional participant was removed from analysis due to excessive amount of artefact in their EEG (more than 30% of the trials were rejected).

### Stimuli

Speech stimuli were 80 pairs of sentences with either an early phrase boundary (EPhB) or a late phrase boundary (LPhB). Some of the sentence pairs were taken from Frazier and Rayner [31], and the remaining were created by a linguist (third author). Both the early and late phrase boundary sentences contained the same words, but the presence of phrase boundary was different between them. For example (# indicates a phrase boundary):

1. Because John studied # the subject matter is clearer now (EPhB).
2. Because John studied the subject matter # it is clearer now (LPhB).

**Figure 1:**
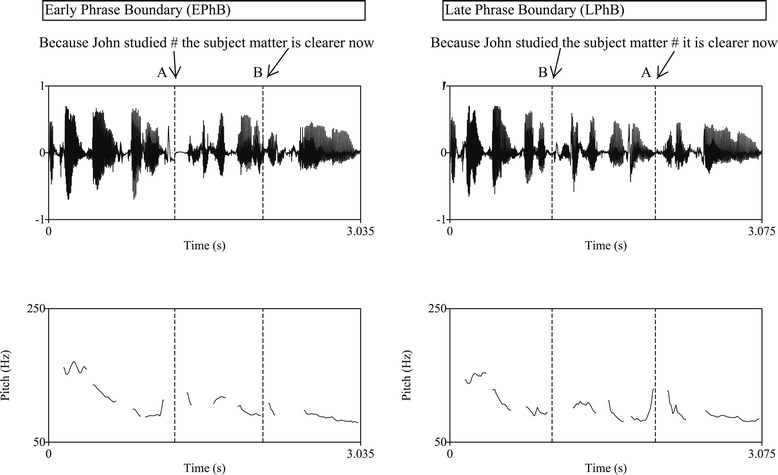
**Waveform and fundamental frequency (F0) contour of example sentences with early phrase boundary (left) and late phrase boundary (right).** The arrows show the place where the additional triggers were placed.

In these sentences, the noun phrase “*the subject matter*” could be either the subject of the second phrase “*the subject matter is clearer now*” (early phrase boundary, example 1) or object of the verb “*studied*” (late phrase boundary, example 2) depending on the position of the phrase boundary. Stimulus example waveforms are shown in Figure 1.

The sentences were spoken by an adult male speaker of Australian English. The speaker did multiple repetitions of the sentences at a normal rate, which were recorded using a unidirectional microphone, and digitized at 44100 Hz with a bit depth of 16. The recorded sentences were analysed using Praat software (Version 5.0.31; [32]) for duration, frequency and intensity measurements. From the pool of 80 sentence pairs, 48 pairs were selected as the stimuli for the present study. This selection was done so that the phrase boundary occurred at approximately the same time for all early phrase boundary sentences (M = 1225 ms, SD = 52 ms) and for all late phrase boundary sentences (M = 1986 ms, SD = 112 ms). None of the sentences were acoustically manipulated, since it would affect the naturalness of the stimuli.

Acoustic analysis data of the pre-boundary syllable was compared with the same syllable in the non-phrase boundary condition. The comparison revealed that the syllable at a phrase boundary was longer in duration [*M* = 363.29 ms (*SD* = 112.83 ms) vs. *M* = 237.72 ms (*SD* = 93.42 ms); *t*(95) = 20.11, *p* = 0.0001], lower in intensity [*M* = 77.92 dB (*SD* = 2.41 dB) vs. *M* = 79.49 dB (*SD* = 2.29 dB); *t*(95) = −7.21, *p* = 0.0001] and was followed by a pause (*M* = 110.63 ms, *SD* = 52.12 ms). The pre-boundary syllable was also characterised by a rise in pitch [*M* = 33.13 Hz (*SD* = 24.66 Hz) vs. *M* = −15.87 Hz (*SD* = 13.37 Hz); *t*(95) = 17.15, *p* = 0.0001] for 146 ms (SD = 111 ms) on average. The acoustic analysis confirmed that the phrase boundary was created by duration, intensity and pitch cues.

Ninety-six filler sentences were created that did not include a phrase boundary. The filler sentences were approximately the same length as the experimental sentences. They were included to prevent any ERP effects resulting from subjects habituating to the same type of sentence structures. The ERP responses to these filler sentences were not analysed. Two examples of filler sentences are given below.

1. The reporters were frustrated by the politician’s answers to their questions
2. The tourists were extremely dispirited before they reached the Himalayas

### Order of presentation

The experimental and filler sentences were presented in pseudo-random order, with the constraint that the same sentence was not presented twice in a row (i.e., one with an early phrase boundary, and one with late phrase boundary). The stimuli were presented diotically via Sennheiser HD 280 Pro headphones (Sennheiser electronic GmbH, Wedemark, Germany). The sentences were presented with an inter-stimulus-interval (ISI, stimulus offset to next stimulus onset) of 2.5 seconds. The stimuli were divided into two blocks of 96 sentences each. Shorter stimuli blocks are recommended for obtaining EEG data with less movement related artifacts [11].

### Recording the electroencephalogram (EEG)

Each participant was seated in a comfortable chair placed 1 m away from computer screen. They were fitted with an electrode cap that held sintered Ag-AgCl electrodes placed at 30 positions on the scalp in line with the 10–20 system (FP1, FP2, F7, F3, FZ, F4, F8, FT7, FC3, FCZ, FC4, FT8, T7, C3, CZ, C4, T8, TP7, CP3, CPZ, CP4, TP8, P7, P3, PZ, P4, P8, O1, OZ, and O2). The ground electrode was positioned between FPz and Fz. Electrical activity was recorded from both the mastoids, with the left mastoid (M1) acting as the online reference. Vertical eye movements (VEOG) were measured with electrodes placed above and below the left eye. Horizontal eye movements (HEOG) were measured with electrodes on the outer canthi of each eye. The electrodes were adjusted until the impedance was below 5 kΩ.

After the electrodes had been fitted, headphones were placed on the participant’s ears, which presented the experimental sentences diotically. The subjects were told that they should ignore the sounds in the headphones, and focus their attention on the silent video on the computer screen. This video did not include subtitles because there is evidence that commas in the written text generate a CPS-like component [13].

While participants were watching the video, and ignoring the sentences, we measured their EEG from each electrode. The signal from the scalp electrodes was amplified 20,000 times (SynAmps 2 amplifier, Compumedics), sampled at 500 Hz, and low-pass filtered at 100 Hz online. This activity was recorded continuously until all stimuli in the two stimulus blocks have been presented.

**Figure 2:**
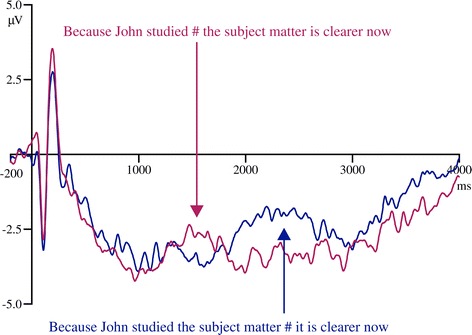
**Grand averaged ERPs for sentences with early phrase boundary (red) and late phrase boundary (blue) at Cz.** ERPs are time locked to the onset of the sentence and epochs cover the whole sentence.

Within each participant’s EEG, the onset of each sentence was marked with a “trigger”. Additional triggers were placed on the experimental sentences to mark (1) the onset of the second phrase in the sentence (see trigger A in Figure 1), and (2) at the onset of the same word in the sentence portion where there is no phrase boundary (see trigger B in Figure 1).

### Processing the EEG

The offline EEG analysis was performed using EEGLAB (http://sccn.ucsd.edu/eeglab/; [33]) and ERPLAB (http://erpinfo.org/erplab/; [34]) toolboxes in MATLAB 2012a (Mathworks, Natik, MA, USA). Portions of the EEG that contained large muscle artefacts were removed from the analysis by visual inspection. The data was then re-referenced to the average of left and right mastoids. The EEG was bandpass filtered using noncausal Butterworth infinite impulse response (IIR) filter with half power cut offs at 0.1 and 30 Hz and a roll of 12 dB/octave. Ocular artefact correction was performed using independent component analysis (ICA) as implemented in EEGLAB (‘eeg_runica’ function). Independent components with known features of eye blinks (based on activity power spectrum, scalp topography, and activity over trials) were identified visually for each participant. The contributions of these components were then removed from the continuous EEG.

In line with previous studies [17,18], the EEG data was processed in two ways. First, we divided the EEG into sections (epochs) that started 200 ms prior to the onset of the first word of the sentence and ended 4000 ms after the first word of the sentence. Each epoch was baseline corrected from −200 to 0 ms. To remove additional artefacts, we used a moving window peak to peak procedure in ERPLAB, with a 200 ms moving window, a 100 ms window step, and a 100 μV voltage threshold. Epochs were averaged separately to produce an ERP for early phrase boundaries and an ERP for late phrase boundaries. Each participant had at least 80% accepted trials per condition (early phrase boundary M = 95.49, SD = 4.67; late phrase boundary M = 94.88, SD = 5.96). The percentage of accepted trials did not differ between experimental conditions (*t*(23) = 0.52, p = 0.61), ensuring no systematic signal to noise ratio differences across conditions. Individual ERP waves were averaged to get grand averaged ERP for each condition. These were our “whole sentence ERPs” for early and late phrase boundaries.

A limitation of this approach is that if a positivity was seen at early or late phrase boundaries, this could reflect (1) a genuine CPS to the phrase boundary, (2) an enhanced N1-P2 response to the onset of the first word in the second phrase which follows a silent pause, or (3) or a combination of both. In order to disentangle the CPS and N1-P2 responses, a second analysis was performed where we divided the EEG into epochs that started 500 ms before the start of the second phrase, and ended 1000 ms after the onset of the second phrase (“phrased ERP”; Trigger A). Similar time intervals were selected for the sentence portion without the phrase boundary (i.e., “unphrased ERP”; Trigger B).This time window allowed us to differentiate the ERP effects that started prior to the onset of the second phrase (−250 ms to 0 ms) where the acoustic cues of phrase boundary are available (e.g., pre-boundary lengthening, pitch change) from the ERPs effects that started after the onset of the second phrase (0 to 250 ms) where the N1-P2 response to the second phrase onset is overlapped with the ERP response to phrase boundary (if any).

Each epoch was baseline corrected from −200 to 0 ms relative to the onset of the sentence. We took the baseline before the onset of the sentence (detached baseline) because the more commonly used immediate baseline (i.e., immediately before the second phrase) is problematic especially for the late phrase boundary: Since the CPS for early phrase boundary happens in the immediate baseline period for the late phrase boundary, there are systematic differences in ERPs in the immediate baseline period. The detached baseline period also has an advantage in excluding the activity related to the last syllable of the pre-boundary word, which contains frequency, intensity and duration cues for phrase boundary.

Epochs with amplitude exceeding 100 μV in a 200-ms moving window with a 100-ms window step were removed from the analysis, and remaining epochs were averaged separately for early phrase boundary and late phrase boundary sentences. More than 80% of the trials were accepted for every participant (early phrase boundary: Trigger-A M = 97.05, SD = 3.52; Trigger-B M = 96.86, SD = 3.79; late phrase boundary: Trigger-A M = 97.31, SD = 5.37; trigger-B M = 97.04, SD = 4.05). A 2 × 2 repeated measures analysis of variance (ANOVA) on percentage of accepted trials with factors boundary type (early, late) and trigger (A,B) showed no significant effect (all F <1, all p > .05), ensuring no systematic signal to noise ratio differences across conditions. Individual ERPs waves were averaged to get grand averaged ERP for each condition. These were our “second phrase ERPs”.

### Measuring the ERPs

Sentence ERPs were measured by computing the mean amplitude in 200-ms time-windows that started at the onset of the phrase boundary. Three time windows were analysed after each phrase boundary (i.e., 1225–1825 ms after the sentence onset for early phrase boundary, and 1986–2586 ms after the sentence onset for late phrase boundary). It is noteworthy that from 1225–1825 ms, the early phrase boundary condition contained a phrase boundary while the late phrase boundary condition did not. Similarly, between 1986–2586 ms, the late phrase condition contained a phrase boundary but the early phrase boundary condition did not. Since early and late phrase boundary sentences contained the same words, the comparison of ERPs in these time windows would reflect the effect of phrase boundary (e.g., early and late phrase boundary ERPs versus unphrased ERPs).

Second phrase ERPs were measured by computing amplitudes for the time window −250 to 0 ms (preceding the second phrase onset) and the time window 0 to 250 ms (following the second phrase onset). A positive response in both windows would suggest that the CPS at the phrase boundary started before the second phrase onset, and later merged with the N1-P2 response to that phrase onset.

### Analysing the ERPs

Separate analyses were done for the whole sentence ERPs and second phrase ERPs. Separate analyses were also done midline and lateral electrode sites. Most of the data sets (around 85%) were normally distributed and followed the assumption of homogeneity of variance. Hence parametric statistics were used to analyse the data. For midline electrodes (Fz, Cz and Pz), a three-way repeated measures ANOVA was performed using the factors boundary type (early, late), condition (phrased, unphrased) and electrode (Fz, Cz, Pz). For lateral sites, electrodes were grouped into four regions of interest (ROIs): right anterior (F4, F8, FC4, FT8), right posterior (P4, P8, CP4, TP8), left anterior (F3, F7, FC3, FT7) and left posterior (P3 , P7, CP3, TP7). A four-way repeated measures ANOVA was performed with the factors boundary type (early, late), condition (phrased, unphrased), hemisphere (right, left) and location (anterior, posterior). These ANOVAs were performed separately for each time interval.

If a significant interaction was found between condition and any other factor, post-hoc one-way ANOVAs were computed to understand the effect of electrode, hemisphere or location for each condition separately. In case of more than one degree of freedom (*df*) in the numerator, Greenhouse-Geisser (G-G) correction was applied to account for potential violations of sphericity. An alpha level of .05 was set as criterion for statistical significance. Partial ŋ^2^ was computed as a measure of effect size.

## Results

We outline below the results for the whole sentence ERPs and the second phrase ERPs separately. Only main effects and interactions involving the factor condition are reported here since they directly related to the aim of the study (i.e., effect of phrase boundaries on ERP).

### Whole sentence ERPs

**Figure 3:**
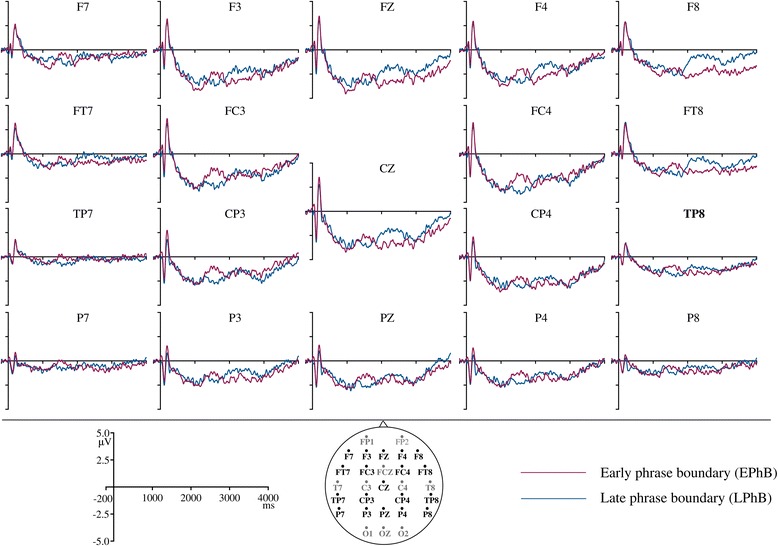
**Grand averaged ERPs for sentences with early phrase boundary (red) and late phrase boundary (blue) at all the electrode sites used for analysis.**

Grand averaged ERPs to early phrase boundary and late phrase boundary sentences at the electrode Cz are shown in Figure 2. The ERPs from all the electrodes used for analysis are shown in Figure 3.

The ERPs to whole sentences with early and late phrase boundaries showed a positive shift starting immediately after the onset of a phrase boundary which lasted around 600 ms. This observation was supported by the statistical analysis. The three-way ANOVA at the midline electrodes revealed a main effect of condition for all three time windows (0–200 ms: F(1,23) = 4.64, p = 0.04, partial ŋ^2^ = 0.17; 200–400 ms: F(1,23) = 18.74, p = 0.001, partial ŋ^2^ = 0.45; 400–600 ms: F(1,23) = 12.76, p = 0.002, partial ŋ^2^ = 0.36). In all time windows, ERPs were significantly more positive for the phrased condition compared to unphrased condition (0–200 ms: phrased M = −2.36, SE = 0.32, unphrased M = −2.90, SE = 0.28; 200–400 ms: phrased M = −2.00, SE = 0.30, unphrased M = −2.98, SE = 0.30; 400–600 ms: phrased M = −2.11, SE = 0.33, unphrased M = −2.95, SE = 0.31). Therefore, the phrase boundaries generated a statistically significant CPS at central electrodes for all time windows.

The four-way ANOVA at lateral electrodes showed a main effect of condition at 200–400 ms (F(1,23) = 19.32, p = 0.001, partial ŋ^2^ = 0.46) and 400–600 ms (F(1,23) = 15.40, p = 0.001, partial ŋ^2^ = 0.40), where the ERPs for phrased condition showed a significantly larger positivity as compared to unphrased condition (200–400 ms: phrased M = −1.19, SE = 0.25, unphrased M = −1.94, SE = 0.25; 400–600 ms: phrased M = −1.28, SE = 0.29, unphrased M = −1.93, SE = 0.24). There was a significant condition × hemisphere interaction between 0–200 ms F(1,23) = 4.25, p = 0.050, partial ŋ^2^ = 0.16. To disentangle the interaction, follow-up one-way ANOVAs were computed for each hemisphere separately with the factor condition. These ANOVAs showed the main effect of condition only at right hemisphere F(1,23) = 4.73, p = 0.04, partial ŋ^2^ = 0.17, where the ERPS were more positive for the phrased condition (phrased M = −1.71, SE = 0.31, unphrased M = −2.17, SE = 0.31).

There was also a significant condition x location interaction between 200–400 ms F(1,23) = 4.62, p = 0.04, partial ŋ^2^ = 0.17. Follow-up one-way ANOVAs conducted at each location showed a main effect of condition at both anterior (F(1,23) = 18.37, p = 0.001, partial ŋ^2^ = 0.44) and posterior locations (F(1,23) = 13.36, p = 0.001, partial ŋ^2^ = 0.37). At both locations, the phrased condition generated a larger positivity compared to the unphrased condition (anterior: phrased M = −1.39, SE = 0.28, unphrased M = −2.32, SE = 0.30; posterior: phrased M = −0.99, SE = 0.24, unphrased M = −1.57, SE = 0.23).

In sum, the analysis on lateral electrodes showed a CPS over right hemisphere between 0–200 ms and at all locations between 200–400 ms and 400–600 ms time intervals. There were no main effects or interactions for boundary type at either midline or lateral electrodes indicating that CPS did not depend on the position of the boundary.

### Second phrase ERPs

**Figure 4:**
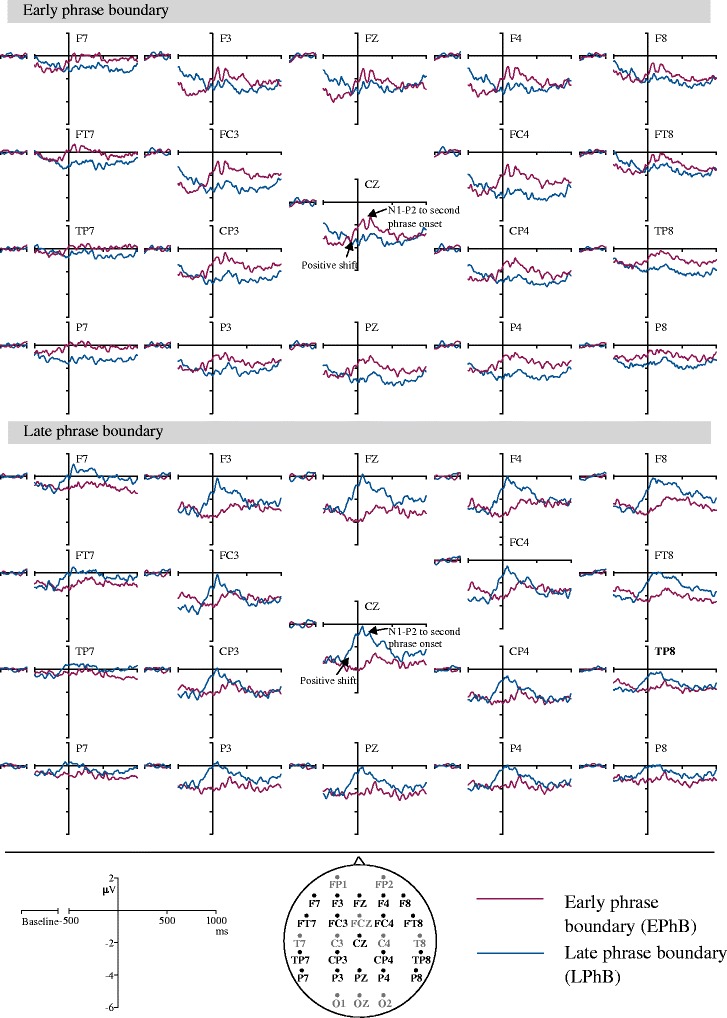
**Grand averaged ERPs time locked to the onset of the second phrase and the corresponding potion without the phrase boundary.**

The ERPs time locked to the onset of the second phrase are shown in Figure 4.

Figure 4 shows that the positivity at the phrase boundary started before the onset of the second phrase and continued after the second phrase onset. This effect was confirmed by the ANOVAs computed for midline and lateral electrodes.

The three-way ANOVA of midline electrodes showed a main effect of condition between for −250-0 ms F(1,23) = 6.89, p = 0.015, partial ŋ^2^ = 0.23; and 0–250 ms F(1,23) = 18.65, p = 0.001, partial ŋ^2^ = 0.45. Phrased stimuli elicited larger positivity compared to unphrased stimuli at both time windows (−250-0 ms: phrased M = −2.21, SE = 0.25; unphrased M = −3.20, SE = 0.34; 0–250 ms: phrased M = −1.16, SE = 0.26; unphrased M = −2.95, SE = 0.33).

The four-way ANOVA at lateral electrodes also showed a main effect of condition for −250-0 ms F(1,23) = 4.41, p = 0.047, partial ŋ^2^ = 0.16; and 0–250 ms F(1,23) = 14.66, p = 0.001, partial ŋ^2^ = 0.39. Similar to the findings at medial electrodes, the phrased condition generated larger positivity at lateral electrodes (−250-0 ms: phrased M = −1.47, SE = 0.21; unphrased M = −2.12, SE = 0.26; 0–250 ms: phrased M = −0.56, SE = 0.22; unphrased M = −1.88, SE = 0.25). Therefore the positivity at the phrase boundaries started before the start of the second phrase and later merged with the N1-P2 responses to the second phrase.

## Discussion

The aim of this study was to use passive ERPs to test if acoustic cues for phrase boundaries in normal English sentences elicit a CPS. We presented 24 English-speaking adults with two types of sentences: sentences with an early phrase boundary, and sentences with a late phrase boundary. We found that both early and late phrase boundaries elicited a significantly more positive ERP from 0 to 600 ms after the onset of the phrase boundary at midline and right hemisphere electrodes, and from 200 to 600 ms at left hemisphere electrodes, than the ERP to the same sentence portion without a phrase boundary.

The positivity seen at phrase boundaries might indicate either an ERP effect for phrase boundary processing (CPS), an enhanced N1-P2 response to the second phrase onset (speech onset following a pause is expected to generate a larger N1-P2 response) or a combination of the two. In order to disentangle the CPS from the N1-P2 response, a second analysis was conducted between −250 ms and 250 ms with respect to the onset of the second phrase [17,18]. This time window allowed us to differentiate the ERP effects that started prior to the onset of the second phrase (−250 ms to 0 ms) where the acoustic cues of phrase boundary are available (pre-boundary lengthening, pitch change) from the ERPs effects that started after the onset of the second phrase (0 to 250 ms) where the N1-P2 response to the second phrase onset is overlapped with the ERP response to phrase boundary (if any). This analysis revealed that the positivity at the phrase boundary started before the second phrase onset and later merged with the N1-P2 response. Therefore, the positive shift observed after the onset of the phrase boundary in the whole sentence analysis does not appear to solely reflect an enhanced N1-P2 response for the onset of speech after a pause. Rather, it contains an additional ERP component possibly reflecting the processing of phrase boundaries in unattended speech. Hence, we call the positivity between 0 to 600 ms in the whole sentence analysis the “passive CPS”.

This is the first study to investigate and show the presence of a passive CPS to unattended normal sentences in adult English speakers. The results confirm that a CPS can be elicited under passive listening conditions, and suggest that acoustic cues that mark phrase boundaries do indeed elicit a CPS in normal English sentences. These findings are similar to previous studies [10,12,13] which demonstrated that the CPS is elicited by phrase boundaries in normal sentences in languages other than English. However, our results differ from these previous studies in three interesting ways. First, the CPS in the current study was present immediately after the onset of the phrase boundary (significant positivity in 0–200 ms window at midline and right hemisphere electrodes). In previous studies, the CPS occurred around 500 ms after the phrase boundary [10,12]. Second, in the current study, the CPS was around 2 μV in size. Only one previous study has reported the amplitude of the CPS (in μV) previously, which was 4 μV [19].

There are two obvious methodological differences that could explain these slight differences in CPS latency and amplitude between studies. First, the previous studies analysed the ERPs in 500-ms time windows, while we analysed the ERPs in smaller 200-ms time windows. Our smaller time window should have allowed a finer analysis of the temporal processing of phrase boundaries, which could reveal different results. Secondly, the current study used a passive auditory paradigm, while the previous studies used an “active” ERP paradigm that asked listeners to actively comprehend the presented sentences. Overt sentence comprehension may trigger additional linguistic processing (i.e., phonemic, syntactic and semantic processing) which may extend up to 600 ms [35]. These additional processes might affect the latency and amplitude of CPS. Hence, the delayed and larger CPS at phrase boundaries in attended sentences in previous studies might reflect both linguistic and acoustic processing, which we wanted to avoid in this study by the passive ERPs. Thus, the CPS in the current study may more closely reflect the acoustic processing of phrase boundaries in speech than linguistic processing of phrase boundaries in speech.

An alternative account for the early passive CPS might be the changes in F0 contour (i.e., pitch) at the phrase boundary. Roll et al. [36,37] found that an increase in pitch of the first word in a phrase generated a relative large P2 response (around 200 ms) in Swedish sentences. In the present study, ERPs were measured to the offset of the final word in a phrase. However the increase in F0 signalling the phrase boundary is present approximately 146 ms before the offset of the final word. It is possible that the positivity observed in the 0–200 ms time window in this study could have contributions from a larger P2 generated by changes in pitch in the last word of the phrase. The similarity between the ERPs to pitch changes in the first word of a phase [36,37] and pitch changes in the final word of the previous phrase (present study) require further investigation.

The third difference between the results of the present study and previous CPS studies is the topography of the CPS. Centro-parietal distribution for the active CPS is previously reported for German sentences [10,12]. Using English sentences, Itzhak et al. [29] reported CPS with fronto-central distribution. Central and right hemisphere distribution of CPS is reported by Pannekamp et al. [10] in the conditions that used jabberwocky sentences, pseudo sentences and hummed sentences. In the present study, we found the passive CPS with central and right hemisphere distribution in the 0–200 ms time window and a broad distribution in the 200–600 ms time windows. Broad distribution of CPS is also reported in active paradigms [15,16]. This suggests that the topography of the CPS is dependent on the stimuli and task. For attended normal sentences, CPS may be triggered by a combination of acoustic and linguistic cues (phonemic, syntactic, and semantic information) in the speech signal. When linguistic cues are reduced, the CPS is broadly distributed, with greater activity in the right hemisphere than left hemisphere.

Interestingly, the location of the phrase boundary did not have an effect on the CPS. Both early and later phrase boundaries elicited CPS with similar magnitude and scalp distribution. This contrasts with the findings of Holzgrefe et al. [16], who found the CPS was seen only for late phrase boundary in German sentences. Differences in stimuli might explain the different outcomes between the studies. The early phrase boundary condition in Holzgrefe et al. [16] inserted a phrase boundary after the first word in the sentence. They attributed the absence of CPS in this “early” phrase boundary condition to two factors: (1) absence of previous prosodic information to process acoustic cues as phrase boundary cues, and (2) an absence of chunking the first word into a larger prosodic unit in the sentences. In the present study the early phrase boundary occurred after three or four words in the sentence (~1 second after the sentence onset). Therefore there was enough prior prosodic information to process the acoustic cues as phrase boundary cues, as well as enough words before the phrase boundary to chunk them together. Hence, a CPS was seen for both early and late phrase boundaries.

## Conclusion

In conclusion, the present study shows that a passive CPS is elicited by acoustic cues that mark phrase boundaries normal English sentences. The timing, amplitude, and topography of the passive CPS are different to the active CPS measured in previous studies. These results show that acoustic cues at the phrase boundary are sufficient to elicit the CPS, and suggest that different processes might be involved in the generation of the CPS in active and passive conditions. The passive CPS that we observed appears to primarily reflect the processing of the acoustic cues in normal English sentences, whereas the active CPS might be reflecting acoustic and linguistic processing.
